# Using “Smart Stimulators” to Treat Parkinson’s Disease: Re-Engineering Neurostimulation Devices

**DOI:** 10.3389/fncom.2012.00069

**Published:** 2012-09-21

**Authors:** Julien Modolo, Anne Beuter, Alex W. Thomas, Alexandre Legros

**Affiliations:** ^1^Bioelectromagnetics, Imaging Program, Lawson Health Research InstituteLondon, ON, Canada; ^2^Department of Medical Biophysics, The University of Western OntarioLondon, ON, Canada; ^3^Bordeaux University, Bordeaux Polytechnic InstituteTalence, France

**Keywords:** mathematical models, neuroengineering, Parkinson’s disease, deep brain stimulation, medical devices

## Abstract

Let’s imagine the cruise control of your car locked at 120 km/h on any road in any condition (city, country, highway, sunny or rainy weather), or your car air conditioner set on maximum cold in any temperature condition (even during a snowy winter): would you find it efficient? That would probably not be the most optimal strategy for a proper and comfortable driving experience. As surprising as this may seem, this is a pretty accurate illustration of how deep brain stimulation is used today to treat Parkinson’s disease motor symptoms and other neurological disorders such as essential tremor, obsessive-compulsive disorder, or epilepsy.

In Parkinson’s disease, DBS is a neurosurgical treatment offered to approximately 5% of patients, and consists in the permanent implantation of stimulating electrodes in deep brain structures (in most cases, the subthalamic nucleus). These electrodes are connected to a pacemaker and deliver electrical impulses at high frequency (typically, 130–180 Hz) 24/7. This treatment drastically relieves tremor, rigidity, and akinesia in most patients. However, DBS sometimes induces adverse effects (e.g., weight gain). Surprisingly, while DBS works well, physiological mechanisms underlying DBS benefits in patients are not known to-date, which constitutes a major drawback.

However, things will change in the near future. Numerous biomarkers (biological indicators of specific physiological or pathological processes, e.g., selected frequency components of brain electrical activity) have been used recently to investigate DBS impact throughout a wide range of brain regions and functions (see Phillips et al., 2006; Bronte-Stewart et al., 2009; Rosa et al., 2010). Although a unified view of DBS impact on brain activity still has to be reached, it is tempting to use such biomarkers as indicators to regulate and optimize “online” the stimulation signal. In other words, monitoring these biomarkers could help to improve DBS by adding a “thermostat” that would “regulate the brain activity” using appropriate stimulation in a smarter way: the brain “temperature” would be monitored and “regulated” only when needed (using relevant biomarkers), and as needed (using relevant stimulation signal). This strategy is termed “closed-loop” stimulation, as opposed to current “open-loop” DBS strategy, i.e., the stimulation signal is fixed over time.

Over the recent years, several research groups have attempted to develop closed-loop neurostimulation methods for Parkinson’s disease, but they are mainly theoretical and essentially exist *in silico* (in computer models) today (Schiff and Sauer, 2008; Modolo et al., 2010). Indeed, innovative closed-loop therapeutics have been mostly developed based on biophysical brain activity models, using mathematical equations describing and predicting neural networks activity. Such models offer a predictive capacity needed to evaluate the brain response under a closed-loop stimulation signal. The common rationale between these closed-loop strategies is to provide a stimulation targeting specific brain areas, to attenuate and control pathological brain rhythms based on instantaneous brain activity recordings. Since these techniques are mainly based on biophysical models rather than animal models, thus involving major simplifications of the real living brain, they understandably give rise to skepticism from the medical community. As a consequence, clinical translation has been limited to-date.

Nevertheless, several groups are developing various techniques aiming “on-demand” brain stimulation techniques. As an example, experimental setups allowing the monitoring of neurotransmitters (e.g., dopamine) during DBS have been developed, intending to evaluate the best moment to deliver electrical stimulation (Roham et al., 2009). The idea that neuromimetic chipsets could be appropriate to provide physiologically relevant stimulation has also been proposed (see the Renachip project, http://www.renachip.org). Furthermore, closed-loop stimulation is already used for cardiac stimulation and implemented in commercial devices (e.g., Medtronic Sensia), with electrical stimulation being delivered as a function of the heart rate. Therefore, closed-loop neurostimulation is no longer a dream, but is it just about translating closed-loop technology from cardiology to neurology? Not only: we think that biophysical models have also their word to say, and that they will accelerate the process. An elegant example is the successful control of neuronal activity by electric stimulation at times predicted by chaos theory (Schiff et al., 1994). This work has provided the “proof of concept” that closed-loop neuromodulation is not only technically feasible, but also that models can assist in this process. Importantly, significant advances in neuroengineering suggest that advanced closed-loop techniques might be available for patients sooner than expected. Indeed, Viventi et al. (2011) have recently proposed a high-density matrix of electrodes flexible enough to adapt to specific gyri and sulci geometry. Such high-resolution spatiotemporal cortical activity mappings, with the capability to record neural signals but also to provide electrical stimulation, represents an extraordinary perspective of new-generation medical devices, of course if, and only if, it is programmed with a “smart” stimulation algorithm. Importantly, encouraging experimental evidence has been provided in a recent study (Rosin et al., 2011) investigating the effects of closed-loop DBS in MPTP monkeys (PD animal model, involving the intake of 1-methyl-4-phenyl-1,2,3,6-tetrahydropyridine – MPTP, destroying dopaminergic neurons and thus mimicking neurodegenerative processes taking place in PD). Indeed, based on stimulation of the GPi (internal segment of the globus pallidus) using a pulse train following spikes detection in GPi or M1 (primary motor cortex), the authors were able to demonstrate an improvement of symptoms (e.g., akinesia). Consequently, there is a convergence of not only theoretical, but also experimental evidence pointing at the use of closed-loop neurostimulation devices in the very near future for the benefit of patients.

Several biophysical models have provided clinically relevant descriptions of *in vivo* human brain activity. For example, neural mass models, consisting of integro-differential equations, accurately describe cortical activity using large assemblies of neurons interconnected with specific types of synapses and associated kinetics. These models have been successful in understanding epileptic onset in humans, and have provided fruitful insights into underlying disease mechanisms (Wendling et al., 2002). Remarkably, they have also been used to successfully describe and predict the occurrence of visual hallucination patterns (Ermentrout and Cowan, 1979), electroencephalographic dynamics activity and its changes during general anesthesia (Hutt, 2011). Since neural mass models are used to describe naturally occurring physiological and pathological phenomena, they are an attractive tool to assist in the conception of closed-loop innovative therapeutic strategies. Thus, in neuroscience and not only in physics; mathematics have an almost insolent success, and we can ask with Albert Einstein (1921, in a lecture to the Prussian Academy of Science in Berlin): “How can it be that mathematics, being after all a product of human thought which is independent of experience, is so admirably appropriate to the objects of reality?” Furthermore, there is increased awareness about the role of dynamical systems theory in our understanding of brain function, as it is emphasized in a recent paper (Potter, 2011): “Perhaps, we are only beginning to appreciate the complexity and dynamics of the neural circuits involved.” Overall, compared to initial models investigating the possibility of closed-loop stimulation in Parkinson’s disease, the use of neural mass and neural field models represents a significant advance in providing biologically relevant description of brain tissue dynamics. By their capacity to simulate electric (electroencephalography) and metabolic (e.g., oxygen consumption as measured by functional magnetic resonance imaging) brain activity, these models bring us closer to meaningful comparisons and validations with experimental data recorded in human patients.

Acknowledging the usefulness of brain activity biophysical models in new therapeutic applications development does not mean abandoning animal or human testing. Indeed, in a solid biologically grounded biophysical model, electrical stimulations like those produced by DBS (e.g., in Parkinson’s disease), can be simulated and integrated to the system of equations. For example, model-based clinical tools have been developed and are used today, in order to optimize DBS parameters to individual patient anatomy and response to stimulation. Such models based on a detailed biophysical description of the interaction between the electric field induced by DBS and the response at the level of nerve fibers make it possible to bridge biophysical models with predictable clinical outcomes (Maks et al., 2009; McIntyre et al., 2011). It becomes then possible to monitor its impact on specific biologically significant outcomes of the model: modulations in the outcomes are indicative of changes in specific physiological parameters (e.g., characterizing neuronal connections architecture). Model results can thus be confronted to experimental results to support existing, or propose new, hypotheses in terms of mechanisms involved. This illustrates how biologically based biophysical brain activity models, such as neural mass models, can improve human data understanding, and should be viewed as a required tool that conveniently and efficiently complements experimental neurostimulation research limitations. In our view, the use of biophysical models will be mandatory to further understand brain activity and to conceive the next-generation of closed-loop neurostimulation devices. Indeed, before talking to the brain, we first have to understand the language it speaks.

A closed-loop neurostimulation therapy, optimized and widely available for patients, is only steps away. It is most likely that biophysical models of brain activity will play a critical role to end this journey: they will help us to escape from current neurostimulation techniques based on random, empirical observations; to propose individualized, smart, and less invasive neuromodulation technologies.

## Conflict of Interest Statement

The authors declare that the research was conducted in the absence of any commercial or financial relationships that could be construed as a potential conflict of interest.
